# Deep Brain Stimulation of the Pedunculopontine Tegmental Nucleus Renders Neuroprotection through the Suppression of Hippocampal Apoptosis: An Experimental Animal Study

**DOI:** 10.3390/brainsci10010025

**Published:** 2020-01-02

**Authors:** Chellappan Praveen Rajneesh, Tsung-Hsun Hsieh, Shih-Ching Chen, Chien-Hung Lai, Ling-Yu Yang, Hung-Yen Chin, Chih-Wei Peng

**Affiliations:** 1 School of Biomedical Engineering, College of Biomedical Engineering, Taipei Medical University, Taipei 11031, Taiwan; pr2502@tmu.edu.tw (C.P.R.); allison73323@gmail.com (L.-Y.Y.); 2 Department of Physical Therapy and Graduate Institute of Rehabilitation Science, College of Medicine, Chang Gung University, Taoyuan 33302, Taiwan; hsiehth@mail.cgu.edu.tw; 3 Neuroscience Research Center, Chang Gung Memorial Hospital, Linkou 33305, Taiwan; 4 Department of Physical Medicine and Rehabilitation, School of Medicine, College of Medicine, Taipei Medical University, Taipei 11031, Taiwan; csc@tmu.edu.tw (S.-C.C.); chlai@tmu.edu.tw (C.-H.L.); 5 Department of Physical Medicine and Rehabilitation, Taipei Medical University Hospital, Taipei 11031, Taiwan; 6 Department of Obstetrics and Gynecology, Taipei Medical University Hospital, Taipei 11031, Taiwan; chin671009@gmail.com; 7 Department of Obstetrics and Gynecology, School of Medicine, College of Medicine, Taipei Medical University, Taipei-11031, Taiwan; 8 Research Center of Biomedical Device, Taipei Medical University, Taipei 11031, Taiwan

**Keywords:** PPTg-DBS, BDNF, caspase-3, lipid peroxidation, hippocampus, apoptosis

## Abstract

The core objective of this study was to determine the neuroprotective properties of deep brain stimulation of the pedunculopontine tegmental nucleus on the apoptosis of the hippocampus. The pedunculopontine tegmental nucleus is a prime target for Parkinson′s disease and is a crucial component in a feedback loop connected with the hippocampus. Deep brain stimulation was employed as a potential tool to evaluate the neuroprotective properties of hippocampal apoptosis. Deep brain stimulation was applied to the experimental animals for an hour. Henceforth, the activity of Caspase-3, myelin basic protein, Bcl-2, BAX level, lipid peroxidation, interleukin-6 levels, and brain-derived neurotrophic factor levels were evaluated at hours 1, 3 and 6 and compared with the sham group of animals. Herein, decreased levels of caspases activity and elevated levels of Bcl-2 expressions and inhibited BAX expressions were observed in experimental animals at the aforementioned time intervals. Furthermore, the ratio of Bcl-2/BAX was increased, and interleukin -6, lipid peroxidation levels were not affected by deep brain stimulation in the experimental animals. These affirmative results have explained the neuroprotection rendered by hippocampus apoptosis as a result of deep brain stimulation. Deep brain stimulation is widely used to manage neuro-motor disorders. Nevertheless, this novel study will be a revelation for a better understanding of neuromodulatory management and encourage further research with new dimensions in the field of neuroscience.

## 1. Introduction

Deep brain stimulation (DBS) is a preeminent modern strategy for various neurodegenerative and cognitive disorders [1]. It involves the distribution of current or an electrical pulse in brain circuits via surgically embedded leads containing electrodes. The application of DBS to specific targets within the brain’s motor circuitry has become a prominent therapy measure for movement disorders [2]. In recent years, due to advancements in surgical procedures, the application of DBS has widened. Hitherto, understanding of the neurophysiological mechanism of DBS is still elusive. DBS is an unavoidable option in the management of neurodegenerative disorders. Typically, DBS is directly connected with neuronal circuits; hence, there are more chances for neuronal loss. In general, neuroprotection can be denoted as the relative preservation of neuronal structure and/or function [3] more precisely defined as decreased neuro-degradation through the reduced central nervous system (CNS) inflammatory activity and new lesion development [4]. The application of DBS is versatile, and it has also exhibited some neuroprotective effects in the brain, to the extent that studies by Du et al. [5] disclosed the neuroprotective effects of subthalamic DBS in rats and monkeys. In Parkinson’s disease patients (PD), DBS enhances neuronal survival through inducing the expression of brain-derived neurotrophic factor (BDNF), which is responsible for the survival of dopaminergic neurons via tropomyosin receptor kinase B signaling [6].

Apoptosis has also been considered as a morphological expression of programmed cell death and has naturally been concomitant with the formation of the typical central nervous system (CNS) during development along with the maintenance of homeostasis of all multicellular organisms [7]. Superfluous oxidative stress kills the cells by inducing apoptosis, and oxidative stress is a biochemical state which is known as an imbalance between the presence of relatively increased levels of toxic reactive species, predominantly comprising of reactive oxygen species (ROS), reactive nitrogen species (RNS), and the antioxidative defense mechanisms [8]. This cell death invariably occurs in the nervous system also apparently, and brain tissues are more susceptible to this [9]. Neuronal death usually occurs at the developmental as well as the differentiation stages. Typically, neuronal death is often protected against by specific neurotrophic factors such as nerve growth factor (NGF), BDNF, neurotrophin (NT-3), and NT-4/5 [10].

The pedunculopontine tegmental nucleus (PPTg) is a brain region that lies between the basal ganglia and brainstem of the brain. It is capable of influencing and regulating all levels of basal ganglia and corticostriatal activity other than being a vital component of brainstem reticular and motor control circuitry. Besides this, PPTg has served as a pivotal target area for DBS for the treatment of several neurodegenerative disorders [11]. The PPTg primarily consists of a subpopulation of coleogenic, glutamergic, and GABAergic neurons in the brain stem [12], and it has been mainly involved in hippocampus formation and theta rhythm. Theta rhythm is a dominant electroencephalographic event that accompanies certain kinds of voluntary movements, attentive immobility, and a paradoxical phase of sleep [13]. Moreover, several research works are being conducted in PPN related to PD and other neurodegenerative disorders. For instance, there is striking evidence revealing that stimulation of the PPTg might have positive effects on Parkinsonian gait disorder, postural instability, and freezing gait [14]. Besides this, PPTg DBS helps in the improvement of cognitive functions in human subjects [15]. For instance, based on recent reports, the neuromodulatory effects PPTg DBS improves memory enhancement in PD and Alzheimer’s disease (AD) patients [1,16]. In our previous study, we investigated the bladder functions of traumatic brain-injured rats in the pontine reticular formation (PnO) with the aid of a DBS strategy [17]. Herein, we found the possibility of PPTg DBS regulation in hippocampal cell survival by showing attenuated effects on apoptosis in the rat hippocampus. Nevertheless, the impact of PPTg DBS on the hippocampus has not yet been investigated extensively. Hence, the purpose of this study was to examine the neuroprotective effects of DBS in the PPTg region via hippocampal apoptosis.

## 2. Materials and Methods

### 2.1. Animal Handling, Surgery, and Imaging Protocols

#### 2.1.1. Animals Selection and Maintenance

Twenty-four adult male Sprague Dawley rats (*n* = 24) weighing between 200–260 g produced from BioLASCO Taiwan, Yilan, Taiwan, were utilized for the present study. All the rats were housed in a well-equipped animal house under an aseptic condition, temperature- humidity-controlled environment according to ethical standards. All the rats were kept under 12:12 h light: dark cycle and had free access to a pellet diet and water ad libitum. The animals were acclimatized for seven to ten days prior to experimental use, and the experimental protocols and animal usage/handling procedures were approved by the Institutional Animal Care and Use Committee of Taipei Medical University (IACUC-TMU-Approval No. LAC 2016-0453).

#### 2.1.2. Grouping and Experiment Schedule of Animals

Rats were divided into four groups (*n* = 6): Group I rats received sham operation and apart from this, other groups such as group II, III and IV received one hour of PPTg DBS. After the DBS procedure, group II rats were sacrificed after an hour; group III rats were sacrificed after 3 h, and group IV rats were sacrificed after 6 h. The group I rats, which received the sham operation, were also sacrificed, and no specific interval was followed for the scarification of this group. To investigate the regulation of anti-apoptotic effects by PPTg DBS, a time-dependent study was performed. Understanding the kinetics of cell death in each model is a critical phenomenon. Especially since some proteins, such as caspases, are expressed only transiently. Apoptotic cells in any system can die and disappear relatively quickly. The entire duration of the apoptotic process can occur rapidly within a few hours. Hence, there may be a chance for false-negative results if the assay is done too early or too late. Besides this, apoptosis can occur at a low frequency or in specific sites within organs, tissues, and cultures. Due to the aforementioned reasons, the present study was designed in a time-dependent manner. Since the present study was an in vivo study, we limited the time and did not exceed 6 h.

#### 2.1.3. Brain Surgery Procedure

The experimental rats were anesthetized using urethane (1.25 g/kg, subcutaneously; Sigma Aldrich Ltd., St Louis, MO, USA) throughout the experiment; since the rats had to be sacrificed at the terminal part of the investigation, a strong anesthetic agent was used. Body temperature was maintained between 36–38 °C by using a recirculating water blanket. The surgical site was shaved, cut, and the midline of the heAl was scalped, and the connective tissue attached to the bone was scraped. Hydrogen peroxide was used to disinfect and clean the skull surface.

#### 2.1.4. Stereotactic Procedures and Deep Brain Stimulation Protocols

Subsequently, the bregma point was exposed under the stereotactic apparatus (Stereotaxic, Stoelting, IL, USA), and the PPTg targeted area was located and marked from the bregma with a deviation of AP −7.3 mm, L +2.0 mm, DV −7.5 mm [16]. Further along, a burr hole was drilled into the bone, and a twisted-wire bipolar stimulating electrode (SS80SNE-100, MicroProbes, Gaithersburg, MD, USA) was implanted into a targeted brain point with the aid of a stereotactic instrument. DBS parameters were 500-μs biphasic rectangular pulses at a 50-Hz frequency; the stimulating intensity was at 2.5 regulated voltage [18]. These stimulation parameters were consistently maintained throughout the experiment only for the experimental animals. The sham group of animals had electrodes implanted but did not receive stimulation. After finishing the stimulation process, the electrode was removed, and the animals were sacrificed for further analysis. Since it was a biphasic and low-intensity pulse, no seizure occurrence was observed in this study.

#### 2.1.5. Magnetic Resonance Imaging to Assess the Deep Brain Stimulation Electrode Location

**Figure 1:**
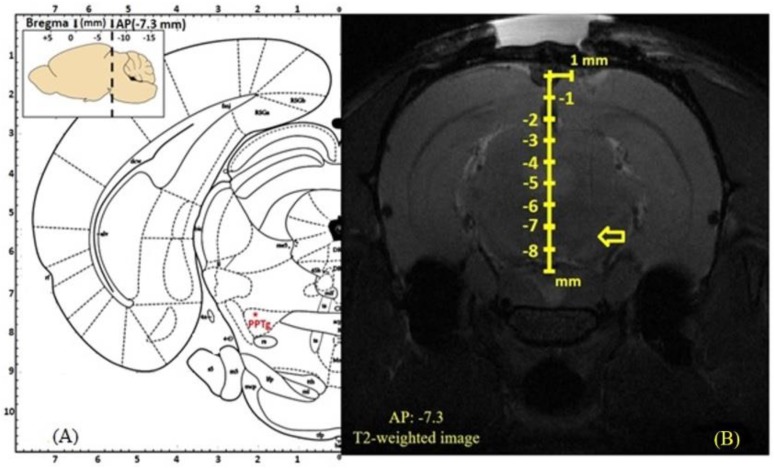
(**A**): The left part shows the schematic representation of the ventral PPTg in an atlas, the red asterisk indicates the ventral point of PPTg (AP 7.3 mm, L +2.0 mm, DV 7.5 mm). (**B**): The MRI results with the tip of a DBS electrode located exactly at the ventral point of PPTg, as indicated by the yellow arrow.

To ratify the DBS target localization of the electrode tip, magnetic resonance imaging (MRI) was implemented. Two live rats (*n* = 2) from each group were selected for the study (group II, III, and IV). Since group I rats did not receive the stimulation, they were excluded from the study. For the MRI experiment, rats were anesthetized with tiletamine zolazepam (50 mg/kg, i.p.) and placed in the prone position in a 7T Bruker PharmaScan 70/160 US (Bruker Medical System, Karlsruhe, Germany). T2-weighted coronal MRI sequences (repetition time/ echo time = 4500/80 ms) were used to localize the tip of the DBS electrode and to avoid the substantial artifact in the MRI result; the metallic DBS electrode was withdrawn from the brain tissue before performing the MRI. The obtained MRI data (Figure 1A,B) were processed by Paravision 6.0 software (Bruker Medical System). As a consequence, our stereotaxic coordination was precisely localized in the target area.

#### 2.1.6. Sacrifice, Removal of Brain Samples and Brain Tissue Collection

For the removal of the brain, animals were euthanized with an overdose of urethane (4 g/kg) by cardiac puncture. Following sacrifice, animals were decapitated by a guillotine, and their brains were carefully removed immediately. After removal, the brains were washed in cold isotonic saline and quickly blotted on filter paper [19]. Different brain regions were collected, including cortex, hippocampus, and brain stem. After collection, the brain tissues were washed with saline, excess saline was removed by tissue paper, and the brain tissues were stored in a −80 °C freezer. Later, the tissues were homogenized in saline (10% *w*/*v*) at 4 °C. The homogenates were centrifuged at 3000 rpm for 10 min in a refrigerating centrifuge, and the supernatant alone was taken for analysis. Caspase-3 activities and lipid peroxidation (LPO) were evaluated in the hippocampus region of the brain, and the Bcl-2, BAX, IL-6, nitrite, and BDNF levels were also determined. Serum was collected, and the myelin basic protein (MBP) levels were estimated.

### 2.2. Biochemical Analysis

#### 2.2.1. Evaluation of Caspase-3 Activity

Caspase-3 activity was determined by using a caspase-3 colorimetric assay kit (Biovisiom, Milpitas, CA, USA). In brief, 50 µL of brain homogenate was added to 96-wells plate and then 50 µL of 2× Reaction Buffer (containing 10 mM DTT) and 5 µL of the 4 mM DEVD-pNA substrate was added to the samples. Then, the plates were incubated at 37 °C for 1–2 h, and the absorbance was measured at 405 nm in an ELISA reader.

#### 2.2.2. Enzyme-Linked Immunosorbent Assay (ELISA)

MBP, Bcl-2, BAX, IL-6, and BDNF levels were quantitatively measured using ELISA kits (Duo-Set; R&D Systems Inc., Minneapolis, MN, USA). For the analysis, the samples were incubated with biotinylated rabbit antibody for 2 h, and then streptavidin-conjugated horseradish peroxidase was added for 20 min. The peroxidase reaction was initiated by adding 3,3′,5,5′-tetramethylbenzidine/H2O2 (R&D Systems Inc., Minneapolis, MN, USA) for 30 min and stopped by adding 0.5 M H2SO4. The absorbance was measured at 450 nm.

#### 2.2.3. Measurement of Lipid Peroxidation Levels (LPO)

Peroxidized lipids were quantified using the lipid peroxidation kit (Calbiochem-Novabiochem Co, Darmstadt, Germany) following the manufacturer’s instructions. For the analysis, 200 μL of the hippocampus tissue homogenate was added with malondialdehyde (MDA) lysis buffer. After that, the lipid peroxidation formed MDA and 4-hydroxynonenal (4-HNE), as natural bi-products. The MDA in the sample reacted with thiobarbituric acid (TBA) to generate the MDA-TBA adduct, and the reaction result was read in the spectrophotometer at 586 nm.

#### 2.2.4. Measurement of Nitrite Concentrations (NO)

For the analysis of nitrite concentrations, the hippocampus tissue was homogenized in Milli-Q water (1:5, *w*/*v*) and then centrifuged (12,000 *g* at 4 °C for 30 min). Nitrite levels were measured after the Griess reaction by incubating 100 µL of tissue homogenate with 100 µL of Griess reagent (Sigma Aldrich Ltd., St Louis, MO, USA) and the sample was read in the spectrophotometer at 550 nm.

### 2.3. Statistical Analysis

Results were presented as the mean ± standard deviation (SD), one-way analysis of variance (ANOVA) followed by Tukey’s honest significant difference method, which was employed to make pairwise comparisons between the sham group and experimental animals. *p* values of <0.05 were considered statistically significant. Statistical analysis was performed using GraphPad Prism 6 software (GraphPad Software, San Diego, CA, USA).

## 3. Results

### 3.1. Effects of PPTg DBS on Hippocampus Caspase-3 Activity

**Figure 2:**
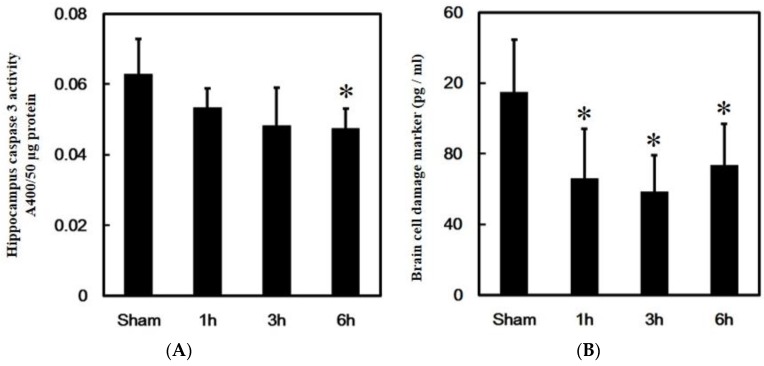
Effects of PPTg DBS on hippocampal cell death. (**A**) Illustration of the apoptosis marker caspase 3 activities evaluated in hippocampal brain tissue. (**B**) Illustration of the brain injury marker MBP levels determined in serum. Both were done at hours 1, 3, and 6-time intervals. Data were presented as mean ± standard deviation (SD) (*n* = 6). Significant differences between groups were analyzed using one-way ANOVA. * *p* < 0.05 was considered statistically significant compared with the sham group.

To investigate the protective effect of PPTg DBS on the hippocampus, apoptosis marker caspase-3 activity, and brain cell damage, marker serum MBP levels were evaluated at hours 1, 3, and 6 after PPTg DBS. Data showed that PPTg DBS had significantly decreased caspase-3 activity at hour 6 of the experimental schedule compared to the sham group (Figure 2A).

Besides this, PPTg DBS had remarkably decreased MBP levels at hours 1, 3, and 6 of the experimental schedule when compared to the sham group (Figure 2B).

### 3.2. Effects of PPTg DBS on Anti- and Pro-Apoptotic Protein Expressions

**Figure 3:**
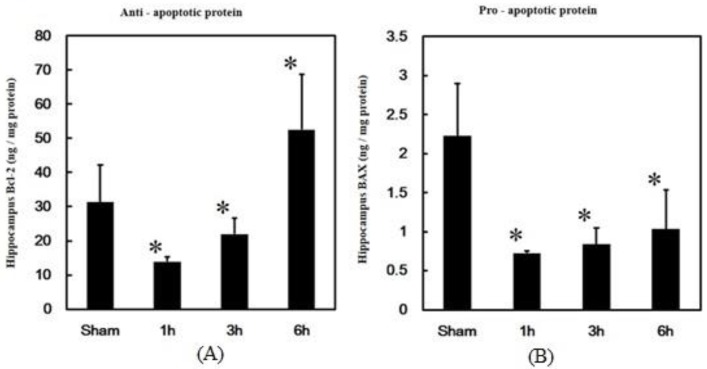
Effects of PPTg DBS on anti- and pro-apoptotic protein expressions. (**A**) Anti-apoptotic protein Bcl-2 expressions and (**B**) pro-apoptotic protein BAX expressions were detected in hippocampal brain tissue at hours 1, 3, and 6- time intervals. Data were presented as mean ± SD (*n* = 6). Significant differences between groups were analyzed using one-way ANOVA. * *p* < 0.05 was considered statistically significant compared with the sham group.

The main regulators of apoptosis in normal animals are the expressions of anti-apoptotic and pro-apoptotic proteins. To further investigate the regulation of apoptosis by PPTg DBS, the expressions of anti-apoptotic protein Bcl-2 and pro-apoptotic protein BAX were examined. The results of the present study showed that PPTg DBS significantly decreased Bcl-2 expressions at hours 1 and 3, but increased at hour 6 of the experimental schedule when compared to the sham group (Figure 3A).

**Figure 4:**
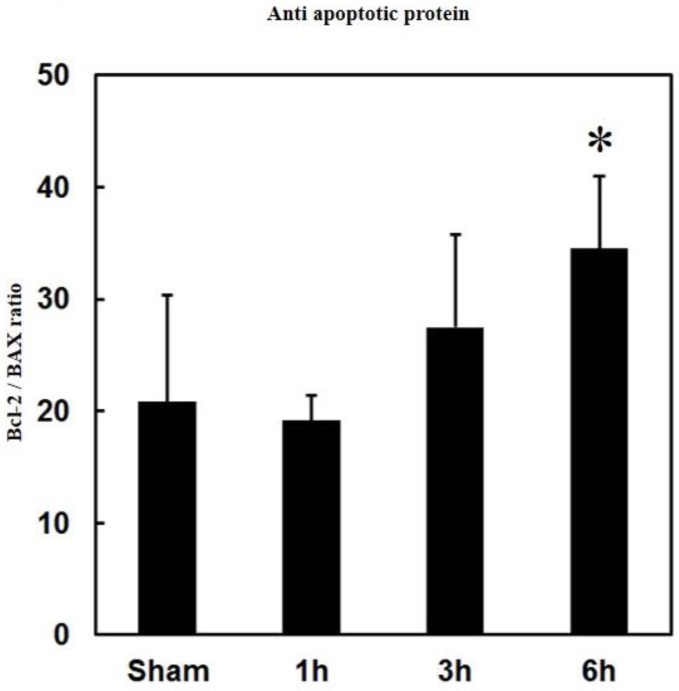
Effects of PPTg DBS on the anti- and pro-apoptotic protein ratio. Illustration of ratio of Bcl-2/BAX expressions were detected in hippocampal brain tissue at hours 1, 3, and 6-time intervals. Data were presented as mean ± SD (*n* = 6). Significant differences between groups were analyzed using one-way ANOVA. * *p* < 0.05 was considered statistically significant compared with the sham group.

PPTg DBS showed significantly decreased BAX expressions at hours 1, 3, and 6 of the experimental schedule when compared to the sham group (Figure 3B). Additionally, the ratio of Bcl-2/BAX significantly increased at hour 6 of the experimental schedule after PPTg DBS (Figure 4).

### 3.3. Effects of PPTg DBS on Hippocampal Inflammation and Oxidative Stress

Inflammation and oxidative stress play a vital role in the initiation process of apoptosis in the hippocampus. To investigate the role of inflammatory regulation in PPTg DBS exerted a protective effect, cytokine IL-6 was analyzed.

**Figure 5:**
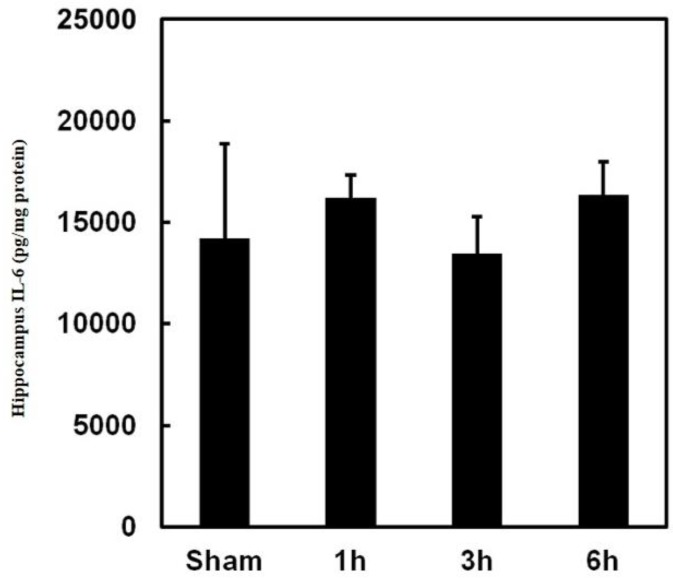
Effects of PPTg DBS on inflammation. Illustration of inflammatory cytokine IL-6 level in hippocampal tissue was determined at hours 1, 3, and 6-time intervals. Data were presented as mean ± SD (*n* = 6). Significant differences between groups were analyzed using one-way ANOVA. * *p* < 0.05 was considered statistically significant compared with the sham group.

The observed data showed that PPTg DBS did not affect IL-6 expression in the hippocampus (Figure 5). To investigate the role of oxidative stress regulation in PPTg DBS exerted protective effect, LPO and nitric oxide levels were evaluated.

**Figure 6:**
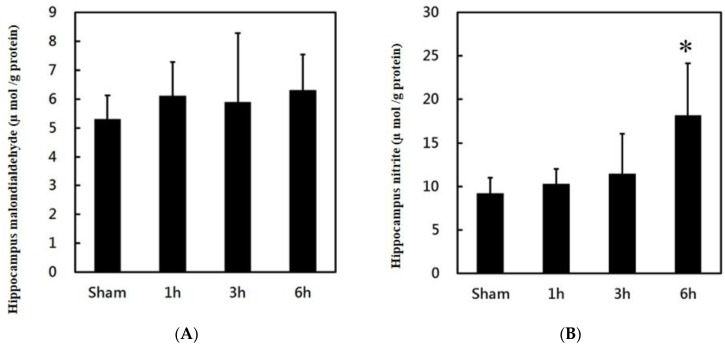
Effects of PPTg DBS on hippocampal oxidative stress: (**A**) illustration of oxidative stress marker malondialdehyde (MDA) and (**B**) illustration of nitrite levels in hippocampal tissue was determined at hours 1, 3, and 6-time intervals. Data were presented as mean ± SD (*n* = 6). Significant differences between groups were analyzed using one-way ANOVA. * *p* < 0.05 was considered statistically significant compared with the sham group.

The obtained data revealed that PPTg DBS did not affect LPO levels in the hippocampus (Figure 6A). However, PPTg DBS significantly increased nitrite levels at hour 6 (Figure 6B) of the experimental schedule.

### 3.4. Effects of PPTg DBS on Neurotrophic Factor BDNF Expressions

Nitric oxide has been reported to be a positive feedback product with neurotrophic factor BDNF production. It is possible that PPTg DBS regulated the BDNF production in the hippocampus of the rats.

**Figure 7:**
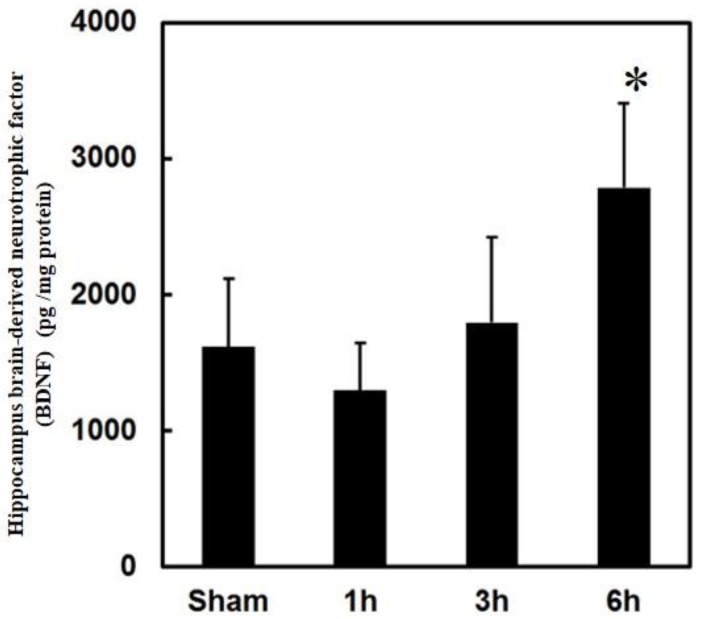
Effects of PPTg DBS on neurotrophic factor brain-derived neurotrophic factor (BDNF) expressions. Illustration of brain-derived neurotrophic factor levels in hippocampal tissue were determined at hours 1, 3, and 6-time intervals. Data were presented as mean ± SD (*n* = 6). Significant differences between groups were analyzed using one-way ANOVA. * *p* < 0.05 was considered statistically significant compared with the sham group.

To investigate the involvement of BDNF in PPTg DBS exerted protective effect, BDNF levels were examined. Observed data clearly showed that PPTg DBS significantly increased the BDNF levels at hour 6 of the experimental schedule (Figure 7).

## 4. Discussion

Apoptosis, the regulated destruction of a cell, is mainly executed by a cysteine protease family. These death proteases are homologous to each other and belong to a large protein family known as caspases [20]. Caspase-3 is typically an indispensable protein for brain development, but it is also a crucial mediator of neuronal apoptosis [21]. Neuronal loss is a common event in many neurodegenerative disorders, and caspase activation appears to be a major execution pathway in physiologic cell death and after moderate insults [22]. Besides this, the MBP level has been suggested to be associated with neurodegenerative disorders, including AD, PD, and neurological insults such as traumatic brain injury [23].

As mentioned earlier, the relationship between the hippocampus and PPTg is mainly associated with the formation of theta rhythm. In animals, theta rhythm has been observed during the interaction between neurons of the medial septum, bundle of Broca, and the hippocampus. In this process, the pontine nuclei play a vital role in the formation of “theta rhythm synchronization system [24]. Besides this, the hippocampus is the most vulnerable region in the brain for inflammation, and increased levels of neuronal apoptosis events have been reported previously in that region [25]. It has been observed that the increased activity of the caspase-3 gene and protein expression is associated with hippocampal apoptosis in mice [26]. In contrast, PPTg DBS significantly decreased caspase-3 activity at hour 6 and MBP levels at hours 1, 3, and 6 in the present study. These results justified the current findings, and DBS itself has neuroprotective effects through reducing hippocampal caspase activity and inflammatory responses by decreasing the glutamate-induced excitotoxicity [27]. Furthermore, the MBP level decreased respectively at hours 1, 3, and 6. Typically MBP can be abundantly found in the cerebrospinal fluid in the condition of brain tissue damage [28]. In the present study, observed BMP levels decreased in turn at hours 1, 3 and 6. Hence, it was evident that PPTg DBS attenuated brain cell death might have protected the hippocampus through inhibiting apoptosis progression.

The intrinsic apoptosis pathway will be activated under cell stress, which is affected by mitochondria permeability. The expressions of pro-apoptotic and anti-apoptotic proteins are the key factors for its regulation. The mitochondria confiscate a concoction of pro-apoptotic protein, including caspases, cytochrome *c*, Apaf-1, AIF, and SMAC/diablo, which would act as an inhibitor for caspase inhibition and exactly how the protein has been released, is enigmatic. In contrast, Bcl-2 family members are actively intricate in the regulation of this process [29]. It has been proven that Bcl-2 inhibits apoptosis and promotes cell survival. Nevertheless, it has also been observed that Bax may contribute to neuronal cell death in AD [30]. The Bcl-2 family principally determines the apoptosis process and BAX; Bak are the main pro-apoptotic proteins. In contrast, the Bcl-2 related anti-apoptotic proteins, such as Bcl-2 and Bcl-xl, may bind and inhibit BAX and Bak, and may directly inhibit Apaf-1-mediated activation of caspase-9 [31]. Additionally, the ratio of anti-apoptotic and pro-apoptotic proteins have been used to evaluate the impact of the apoptosis response [32].

Typically, elevated levels of Bcl-2 facilitate the blocking of apoptosis by preventing the activation of the caspase cascade, blocking the loss of the mitochondrial membrane potential, and inhibiting the release of cytochrome *c* from mitochondria [33]. Besides this, overexpression of BAX homodimers (BAX/BAX) can create large pores in the outer membrane of mitochondria and persuade apoptotic cell death through cytochrome *c*. However, BAX will be neutralized when heterodimerized with Bcl-2 protein family (Bcl-2/BAX), and these heterodimers prevent pore formation. Hence, apoptosis will be inhibited by Bcl-2, which acts as a suppressor of cell death [34]. In the present study, the observed results demonstrated that Bcl-2 expression was upregulated, and BAX expression was down-regulated at hour 6 in PPTg DBS rats; hence, the expression of BAX might be suppressed by the heterodimers of Bcl-2. In addition, the ratio of Bcl-2/BAX was significantly elevated by PPTg DBS. The elevated levels of Bcl-2/BAX ratio appear to be a response that would be protective against apoptosis [34]. Hereafter, it is suggested that the regulation of pro-and anti-apoptotic protein is involved in the anti-apoptotic effect of PPTg DBS.

Inflammation and oxidative stress are significant in regulating the pro-apoptotic and anti-apoptotic protein expressions. Inflammatory cytokines have been reported to regulate apoptosis and Bcl-2 and BAX protein expressions [35]. IL-6 is considered to be one of the first cytokines that appears during an inflammatory response, and also regulates the expressions of Bcl-2 and BAX. Neurodegeneration is mainly facilitated by several inflammatory and neurotoxic mediators such as interleukins, necrosis factors, reactive oxygen species (ROS), reactive nitrogen species (RNS), and mitogen-activated protein kinases (MAPKs) [36]. It has been reported that, in the CNS, microglia are the prime source of ROS through intracellular peroxidase, oxidative processes in mitochondria, and NADPH oxidase activity at the cell surface and membrane [37]. Microglia are activated by ROS that accumulates oxidative stress; the activation of microglia itself can further enhance ROS production [38]. Excessive free radical generation contributes to the pathogenesis of many diseases, and it also damages the DNA structure. It also destroys the cell and leads to the loss of neurons, and it has been documented that the prolonged presence of high levels of reactive oxygen radicals in tissues accelerates the risk for neurodegeneration [39]. The literature reports have resolutely stated that microglial accumulation and activation occur in sites where neurons eventually die and are lost [40].

In the present study, PPTg DBS did not affect IL-6 and LPO levels in the hippocampus. The recent reports have shown that the cytokines were constitutively expressed in CNS, performing as a modulator of neural functions and neuronal survival [41] and that cytokines might not be dysfunctioned by chronic stress treatment such as PPTg DBS. However, PPTg DBS significantly increased the nitrite levels at hour 6. The rate of lipid peroxidation intensely hinges on the concentration of peroxyl radicals. Conversely, numerous experiments have demonstrated that nitric oxide is a potent inhibitor of the propagation reaction in the process of lipid peroxidation [42,43]. Hence, in the present study, it was possible that the increased levels of NO might have played an offensive role in the suppression of LPO. Besides this, similar types of protective effects of NO were also documented in the studies of atherosclerosis [44].

BDNF is one of the neurotrophic factors which acts on certain neurons of the central nervous system and the peripheral nervous system, aiding in the survival of existing neurons and boosting the growth and differentiation of new neurons and synapses. It is highly active in the hippocampus, cortex, and basal forebrain areas, which is vital for learning, memory, and higher thinking [45]. BDNF is commonly expressed in the CNS, retina, kidney, saliva, prostate, motor neurons, and skeletal muscle. Reports state that decreased levels of BDNF are associated with numerous neurodegenerative disorders such as PD, AD, multiple sclerosis, and Huntington’s disease [46]. In the present study, the obtained data clearly showed that PPTg DBS significantly increased the expressions of BDNF in the hippocampus. Increased levels of BDNF induce an increased level of neuroprotection through actively suppressing the expression of caspase-3. Since caspase-3 turns out to be the decisive executioner of apoptotic death in neurons, regulation of its activity acts as a dominant tool in controlling neuronal cell survival [47]. Remarkably, decreased activity of caspase-3 was also observed in the present study. Hence, it serves as evidence that BDNF might render neuroprotection for hippocampus survival.

It has been documented that chronic DBS can induce the progressive recognition of neuronal circuits over enhanced synaptic plasticity and neurogenesis. Moreover, a recent investigation in preclinical animal models and humans put forward that DBS may protect neurons from disease-related neurotoxicity in a certain set of circumstances [48]. PPTg is one of the prime targets for PD patients to improve motor function. PD’s motor dysfunction is due to the death of midbrain dopamine-generating cells in the substantia nigra [40]. Besides this, neuroprotective effects have been chiefly documented in PD patients in the process of STN DBS [49]. The reference above raises a tremendous possibility that DBS is certainly involved in neuroprotection and neuronal survival. To magnify the therapeutic benefits and to avoid side effects, specific control over the stimulation field is necessary. Above and beyond this, harnessing the effects of DBS is dependable on spatial dispersal of the targeted area according to brain anatomy [50]. In the present study, PPTg DBS inhibited hippocampus activity and increased neurotrophic factor BDNF levels. Substantial evidence has been shown that the hippocampus was involved in the process of neuroprotection after the PPTg DBS. However, the effect of PPTg DBS on the hippocampus in the rat model still requires in-depth investigation.

## 5. Conclusions

This study demonstrated the protective effect of PPTg DBS on hippocampus survival in the rat brain. The results of the present experiments revealed that the PPTg DBS significantly inhibited caspase-3 activity, decreased brain cell damage marker BMP level, and increased the ratio of Bcl-2/BAX. Nevertheless, the levels of the inflammatory cytokine IL-6 and oxidative stress marker LPO were not affected by PPTg DBS. In contrast, PPTg DBS significantly increased the levels of nitric oxide and neurotrophic factor BDNF in the rat hippocampus brain region. Hence, we have suggested that PPTg DBS has the potential to enhance hippocampal cell survival through increasing BDNF production in the hippocampus region.
